# A pilot study of cervicovaginal microbiome patterns associated with embryo implantation outcomes in endometriosis-associated infertility

**DOI:** 10.3389/fmed.2025.1642770

**Published:** 2025-12-18

**Authors:** Yuhong Li, Dandan Chen, YangKun Feng, Qiuping Li, Wei Mao, Ping Yu, Yun Zhang

**Affiliations:** Affiliated Wuxi Maternal and Child Health Hospital, Wuxi School of Medicine, Jiangnan University, Wuxi, China

**Keywords:** endometriosis, assisted reproduction, frozen embryo transfer, cervical microbiota, vaginal microbiota, 16S rDNA, infertility

## Abstract

**Background:**

The cervicovaginal microbiome—spanning from the vagina to endometrium—remains undercharacterized in endometriosis-associated infertility. Objective: To determine whether combined vaginal and cervical microbial profiles predict frozen embryo transfer (FET) outcomes.

**Methods:**

In 22 endometriosis patients undergoing FET, paired vaginal and cervical samples were collected on transfer day. 16S rDNA sequencing quantified microbial composition; alpha/beta diversity, PCoA, LEfSe, and PICRUSt analyses identified taxonomic and functional signatures linked to implantation success. Conduct a differential analysis of microorganisms in different body parts through DMI.

**Results:**

Microbial profiles associated with successful pregnancies featured a higher relative abundance of *Lactobacillus* and *Bifidobacterium*, whereas *Gardnerella, Streptococcus*, and *Atopobiu*m were more enriched in failures. Cervical alpha diversity was significantly lower in successful transfers. LEfSe highlighted differential taxa including *Peptostreptococcales* in successes and *Pseudomonadaceae* in failures. Functional inference predicted dysregulated metabolic pathways in failure-associated communities. Furthermore, the cervical microbiota exhibited higher DMI, indicating greater individual specificity.

**Conclusions:**

Our pilot findings suggest that a continuous cervicovaginal microbial ecosystem presents distinct taxonomic and functional patterns associated with FET success in endometriosis. Specifically, cervical microbiota profiling emerges as a promising, minimally invasive approach worthy of further investigation to potentially personalize ART strategies.

## 1 Introduction

Endometriosis (EM) is one of the common diseases in women of reproductive age, which refers to the appearance of endometrioid tissues (including glands and stroma) in other parts of the uterus outside the uterus. Among these patients, the incidence of infertility is about 40%−50% (1). Studies have shown that women with endometriosis are about 20 times more likely to develop infertility than women without the disease (2, 3). Treatment options for endometriosis with infertility are diverse. Medication can relieve some symptoms, but it is difficult to completely cure. Surgery is the preferred way to remove lesions and improve fertility, but its effect on improving fertility is limited and the recurrence rate is high. Assisted Reproductive Technology (ART) provides an effective alternative to achieve pregnancy (4). The 2022 (5) European Society Human Reproduction Embryology (ESHRE) guideline proposes that: ART can be used to treat infertility associated with endometriosis, especially in cases of impaired fallopian tube function, presence of male infertility factors, low endometriosis fertility index (EFI), and other treatment failures. The incidence of infertility in patients with endometriosis is significantly higher than that in the general population, and the reasons may involve a variety of factors, including abnormal immune function, inflammatory response, hormone level imbalance, and impaired endometrial receptivity. Despite technical advances in ART, implantation failure remains a major obstacle in endometriosis-associated infertility, suggesting that factors beyond embryo quality—particularly the uterine microenvironment—are critical determinants of success.

Traditionally considered sterile, the upper reproductive tract now is recognized to host diverse microbial communities that influence gynecological health. This continuum of microbial niches dynamically interacts with mucosal immunity, epithelial barrier function, and endocrine signaling, influencing implantation and early pregnancy (6–8).Vaginal dysbiosis, characterized by depleted Lactobacillus and overgrowth of anaerobic bacteria, correlates with adverse reproductive events including bacterial vaginosis, preterm birth, and implantation failure (9). Cervical microbiota, positioned at the interface of vaginal and endometrial ecosystems, may more directly reflect endometrial receptivity (10, 11).

Emerging evidence suggests that the cervical microbiota may more accurately mirror endometrial conditions than the vaginal microbiome, as it lies at the transitional interface between the lower and upper reproductive tracts (12). However, previous studies have predominantly assessed single sites (either vaginal or cervical) and have rarely focused on the integrated cervicovaginal ecosystem in the specific context of endometriosis.

Furthermore, endometriosis is characterized by immune dysregulation and heightened oxidative stress, which may alter microbial composition and metabolic output—particularly short-chain fatty acids, lactic acid, and tryptophan metabolites—thereby reshaping endometrial receptivity. Yet, the spatial stability and individuality of microbial profiles within the cervicovaginal continuum in these patients remain poorly defined.

Prior studies have largely examined vaginal or cervical microbiota in isolation, often in heterogeneous infertility populations. In endometriosis-associated infertility—a subset with distinct immunological and inflammatory profiles—the integrated cervicovaginal microbiome remains unexplored. The cervix, positioned at the interface between the vaginal and endometrial ecosystems, may provide a more direct and accessible reflection of the upper reproductive tract's state than vaginal samples alone (13). Therefore, this pilot study aimed to investigate the integrated cervicovaginal microbiome in a homogeneous cohort of endometriosis patients. We hypothesized that distinct microbial patterns at this interface are associated with implantation outcomes. Understanding these associations could generate novel hypotheses and inform future research into biomarkers to optimize FET outcomes.

## 2 Objects and methods

### 2.1 Subjects

A total of 22 patients who underwent Frozen Embryo Transfer (FET) due to endometriosis combined with infertility in Reproductive Medicine Center Hospital of Wuxi Maternal and Child Health Hospital from June 2023 to May 2024 were included in this study. The patients were divided into the following two groups according to the pregnancy outcome: 11 cases in the successful pregnancy group and 11 cases in the failed transplantation group. The vaginal samples from the successful pregnancy group were labeled Y1, and the cervical samples from this group were labeled S1. The vaginal samples from the transplant failure group were labeled Y2, and the cervical samples from this group were labeled S2. A total of 44 samples were collected. Vaginal and cervical secretions from all patients were collected on the day of transplantation, and patients confirmed no antibiotic or vaginal probiotic use within 1 month, ensuring no direct antimicrobial effects on the sampling day.

Inclusion criteria (each item needs to be met): (1) 20 < age < 40 years; (2) 18.5 < BMI < 23.9; (3) Stage III or above EM patients confirmed by laparoscopic surgery and pathological examination; (4) women of childbearing age who did not use contraception and were not pregnant for more than 1 year and had normal sexual life; (5) no use of antibiotics within 1 month; (6) At least one good-quality embryo (grade I-II D3 embryo or AA, AB, BB D5/D6 blastocyst) was included in the transferred embryo. This study did not conduct preimplantation genetic testing (PGT) on the transferred embryos. All the transferred embryos were evaluated as high–quality blastocysts based on morphological criteria (e.g. Gardner score).Exclusion criteria: (1) patients diagnosed with premature ovarian failure, polycystic ovary syndrome, hyperprolactinemia, diabetes mellitus and other endocrine and metabolic diseases; (2) uterine organic diseases: such as benign and malignant uterine tumors, endometrial polyps, etc. (3) Malformation or abnormality of uterus and reproductive organs: septate uterus, bicornuate uterus, etc. (4) To minimize confounding effects on the microbiota, patients who had used vaginal probiotics or antibiotics (systemic or local) within 1 month prior to enrollment and during the transplantation cycle were excluded.

All experiments in this study were conducted under the ethical approval guidelines of the National Research Council, and have been reviewed and approved by the medical ethics committee of the hospital.

### 2.2 Specimen collection

On the day of transplantation, vaginal and cervical sampling was performed before irrigation and transplantation. The patient took the lithotomy position, wore sterile gloves, slowly dilated the vagina with a vaginal dilator, collected the secretions from the vaginal wall of the patient, and after sampling the vaginal secretions, the vaginal dilator gently exposed the cervix, and another sterile swab was taken 2–3 cm into the cervix. After sampling, the cervix was gently exposed by the vaginal dilator, and another sterile swab was taken 2–3 cm deep into the cervix. (The cervical secretion was only in the cervical canal, and the vagina was not touched). Remove the swab. Samples were labeled, followed by rapid freezing in liquid nitrogen and storage at −80 °C before PCR amplification and sequencing.

### 2.3 16s rDNA sequencing and amplification

DNA was extracted with D3142 kit (Guangzhou Meji Biotechnology Co., LTD., China). DNA concentration and purity were detected by NanoDrop 2000 Nanorop microspectrophotometer (Thermo Fisher Scientific USA, Inc.), and the quality of DNA extraction was detected by 1% agarose gel electrophoresis. The V3-V4 variable region was amplified by PCR using 341F (CCTACGGGNGGCWGCAG) and 806R (GGACTACHVGGGTATCTAAT) primers. PCR products were examined by electrophoresis on a 2% agarose gel. Sequencing libraries were constructed using the Illumina DNA Prep Kit (Illumina, CA, USA) and the ABI StepOnePlus Real-Time PCR System (Life Technologies), the library quality was tested by Novaseq 6000 PE250 mode pooling for sequencing (NovaSeq6000 S2 Reagent Kit v1.5, Illumina, USA).

### 2.4 Degree of microbial individuality (DMI)

Degree of microbial individuality (DMI) was measured as the mathematical difference between a given genus regarding their populational median of the inter-individual BC distance and the median of the intra-individual BC distance. To assess the robustness and variability of the DMI estimates, we employed a bootstrap resampling technique. For each genus, we resampled the data with replacement and computed the DMI using the following formula:

DMIi = BC inter - individual - BO intra - individual.

### 2.5 Bioinformatics analysis

Bioinformatics analysis Based on the results of species annotation, the sample Shannon index and Simpson index were calculated to analyze the α diversity of bacterial microbiota. principal component analysis (PCoA) based on Bray-Curtis distance was used to evaluate the β–diversity of the microbiota. To mitigate the potential impact of contamination in this low-biomass study, a rigorous bioinformatic contamination removal process was applied post-sequencing. Briefly, the decontam package (v1.20.0) in R was used with the “prevalence” method (threshold 0.5) to identify and remove sequences likely originating from contaminants. Additionally, a strict prevalence filter was applied, removing any Amplicon Sequence Variant (ASV) that was not present in at least 10% of the samples within any single study group. The results of species annotation were combined at the phylum and genus levels, respectively, and all the bacteria that were not classified into a phylum or genus were classified as unclassified. The top 10 bacterial groups with the highest relative abundance were retained and the remaining bacterial groups were combined and represented by others to draw the stacked bar chart. Wilcoxon rank sum test was used to analyze the top 10 bacterial genera with the mean relative abundance of the two groups. LDA effect size (LEfSe) was used to screen the bacteria with significant differences at each level with LDA > 1.5 as the criterion.

### 2.6 Statistical analysis

The nonparametric Mann-Whitney *U* test was used to compare differences in medians and Fisher's exact test was used to assess differences in proportions. Overall significance was assessed by the Kruskal-Wallis test and chi-square test, and significant results were further compared in pairs. Given the pilot and exploratory nature of this study with a modest sample size, the primary focus was on identifying large effects and generating hypotheses. While the sample size limits the power to detect small effects, the significant differences observed (e.g., in alpha diversity) suggest that the study was adequate for detecting the substantial effects present in this specific, homogeneous cohort. All *P* values were two-sided, and the significance level was set at 0.05. Statistical analyses were performed with the use of R with SPSS, version 22.0.

## 3 Results

### 3.1 Clinical characteristics

Comparison of clinical characteristics and general data between the successful pregnancy group and the transplantation failure group. There were no statistically significant differences in the distribution of age, body mass index (BMI), anti-Müllerian hormone (AMH), follicle-stimulating hormone (FSH), estradiol (E2), luteinizing hormone (LH), prolactin (PRL), testosterone (T), and clinical characteristics between the two groups (*P* > 0.05), indicating comparability (Tables 1 and 2).

**Table 1:** Comparison of the general situation of patients with successful pregnancy and transplant failure.

**Characteristics**	**Successful pregnancy (*n* = 11)**	**Transplant failure (*n* = 11)**	***t***	***P*-value**
Age	31.91 ± 2.95	33.82 ± 3.66	−1.348	0.193
BMI	21.01 ± 2.81	22.41 ± 1.64	−1.428	0.169
AMH	4.63 ± 4.35	3.70 ± 2.18	0.637	0.531
FSH	8.92 ± 3.71	7.97 ± 2.12	0.743	0.466
E2	22.84 ± 5.91	26.56 ± 7.85	−1.258	0.223
LH	4.59 ± 2.85	3.98 ± 1.52	0.634	0.533
PRL	12.69 ± 5.56	15.74 ± 5.58	−1.287	0.213
*T*	0.36 ± 0.18	0.38 ± 0.11	−0.375	0.711
Intimal thickness at conversion day	9.73 ± 2.06	8.50 ± 1.01	1.771	0.092
Excellent embryo rate	0.95 ± 0.15	0.91 ± 0.20	0.598	0.557
Previous ET	0.55 ± 0.93	0.55 ± 0.82	0.000	1.000
Number of transplants	1.27 ± 0.47	1.18 ± 0.40	0.488	0.631

^*^P < 0.05, ^**^P < 0.01.

**Table 2:** Comparison of clinical characteristics of patients with successful pregnancy and transplant failure.

**Characteristics**	**Successful pregnancy (*n* = 11)**	**Transplant failure (*n* = 11)**	***P*-value**
**Presence or absence of vaginal irritation within 24 h**			
Have	0 (0.0)	0 (0.0)	1.0
No	11 (100.0)	11 (100.0)	0
**Presence or absence of vaginal itching within 24 h**			
Have	0 (0.0)	0 (0.0)	1.0
No	11 (100.0)	11 (100.0)	0
**Presence or absence of abnormal discharge within 24 h**			
Have	0 (0.0)	0 (0.0)	1.0
No	11 (100.0)	11 (100.0)	0
**Any vaginal burning within 24 h**			
Have	0 (0.0)	0 (0.0)	1.0
No	11 (100.0)	11 (100.0)	0
**Whether you have painful urination within 24 h**			
Have	0 (0.0)	0 (0.0)	1.0
No	11 (100.0)	11 (100.0)	0

### 3.2 Microbial diversity

According to the Wilcoxon test, only the cervical microbial community Shannon index (*P* = 0.043) and Simpson index (*P* = 0.0431) showed statistically significant differences between the successful pregnancy group and the failed embryo transfer group. However, no significant difference was observed in the alpha diversity analysis of vaginal microbiota (*P* > 0.05). This finding suggests that the microbial diversity of the cervical microecological environment may be more relevant to the success rate of embryo implantation than the vaginal microbiota.

**Figure 1:**
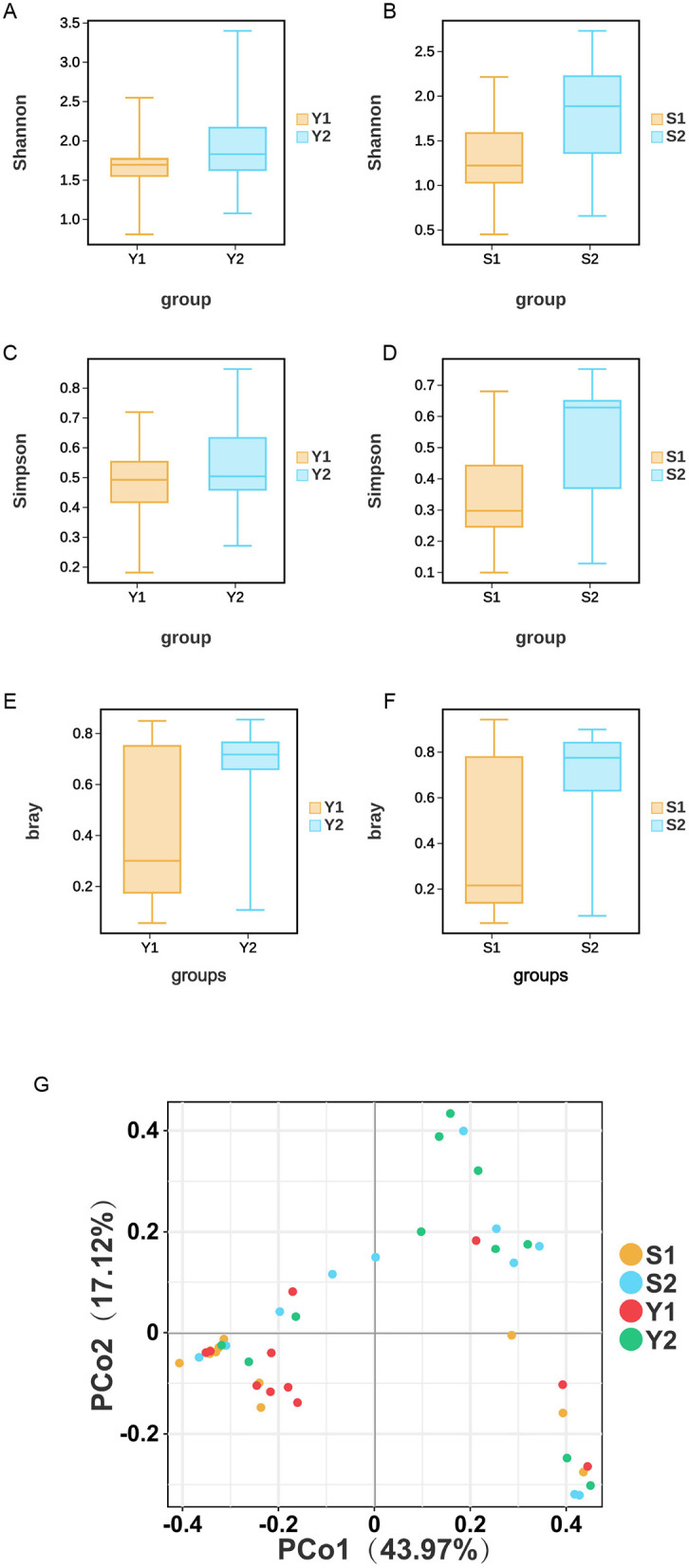
Microbial diversity. **(A)** Differences in vaginal Shannon index. **(B)** Differences in vaginal Simpson index. **(C)** Differences in cervical Shannon index. **(D)** Differences in cervical Simpson index. **(E)** Vaginal β-diversity. **(F)** Cervical β-diversity. **(G)** PCoA.

According to Welch's *t*-test at OTU level, there was a significant difference in the β diversity of cervical and vaginal microbiota between the two groups. It is noteworthy that the β-diversity index of the patients in the failed embryo transfer group was significantly higher than that in the successful pregnancy group, suggesting that the vaginal and cervical microbial communities of the patients in the failed embryo transfer group may have a higher diversity of microbial composition. This finding suggests that lower β-diversity or a more stable microbiota structure may be potentially associated with successful pregnancy. The results of PCoA showed statistically significant differences in the distribution of vaginal and cervical microbiota. as shown in Figure 1.

### 3.3 Differential analysis of biomarkers

**Figure 2:**
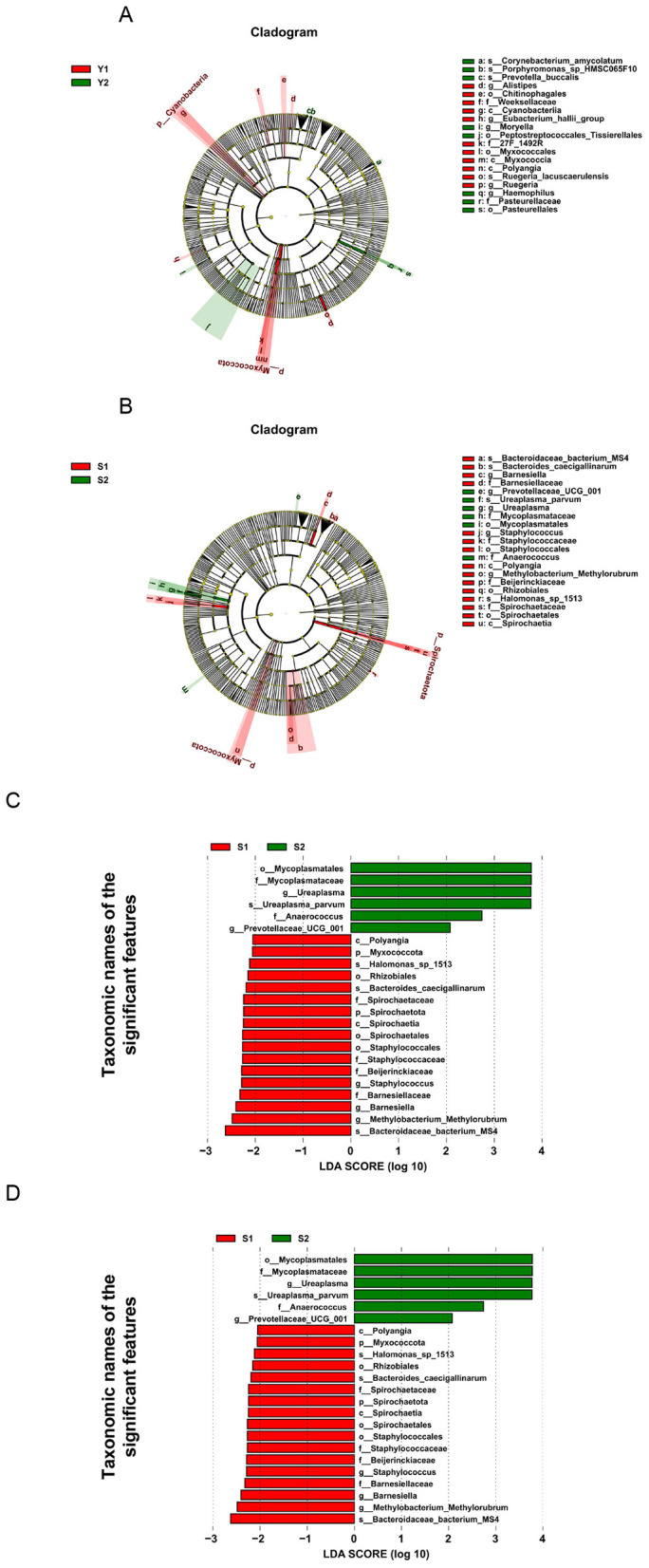
LEfSe analysis. **(A)** Vaginal LDA diagram, with LDA > 1.5; **(B)** Cervical LDA diagram, with LDA > 1.5; **(C)** community distribution map of vaginal species tree; **(D)** community distribution map of cervical species tree.

**Figure 3:**
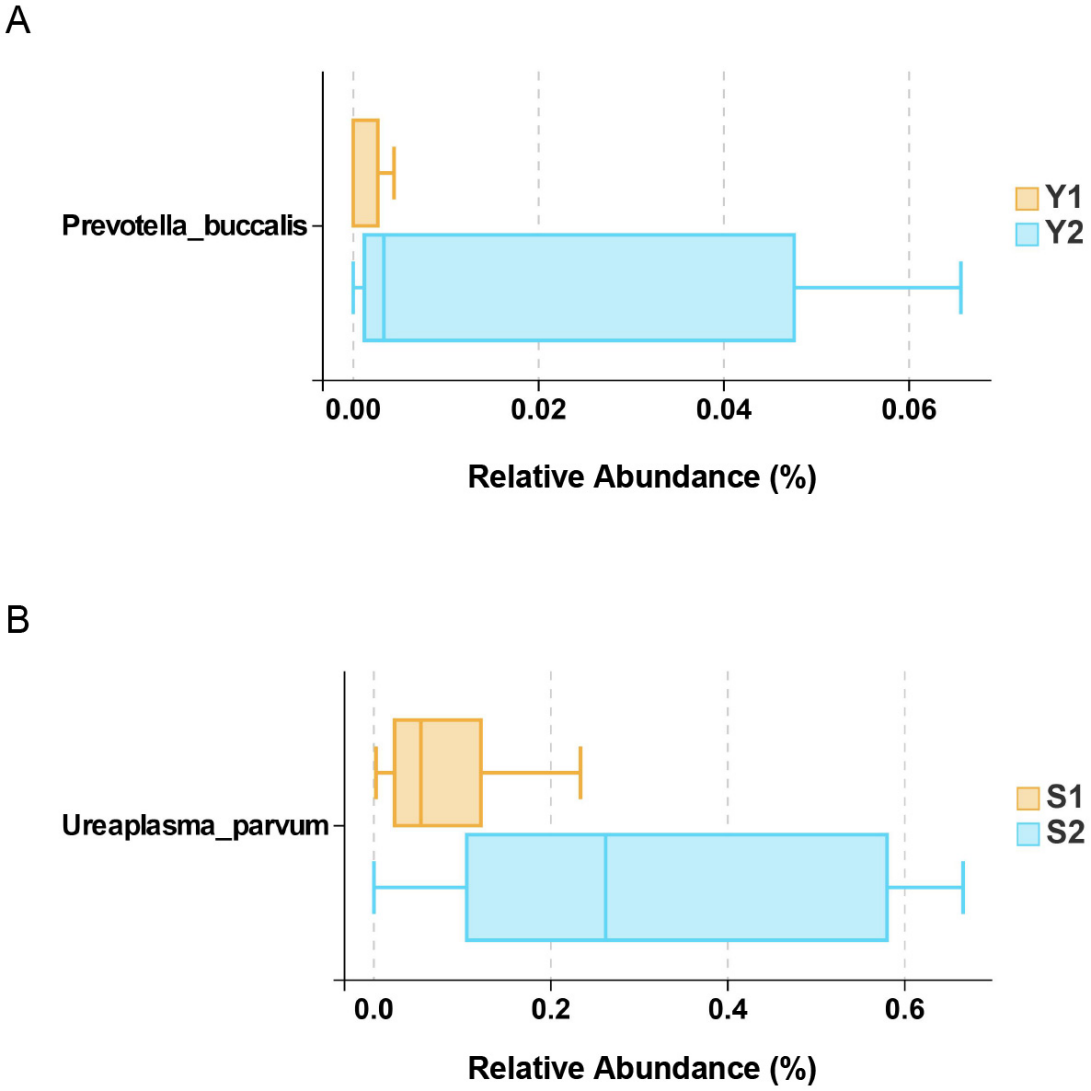
Vaginal and cervical differential microbiota. **(A)** Vaginal microbiota; **(B)** cervical microbiota.

Comparative analysis showed that there were significant differences in microbial composition between the successful pregnancy group and the transplant failure group. In vaginal microbiota, *Eubacterium halli* and *Myxococcales* were more abundant in successful pregnancy group. *Peptostreptococcales-tissierellales* and *Haemophilus* were the main bacteria in the vagina of patients with transplant failure. In the cervical microbiota, *Bacteroidaceae bacterium Ms4* and *Methylobacterium* were the dominant bacteria in the cervix of successful pregnancy patients (Figure 2). And transplant failure in patients with cervical littered showed higher *Mycoplasmatales* and *Mycoplasmataceae* such as abundance. Further analysis at species level by Wilcoxon rank sum test revealed a significant difference between *Prevotella buccalis* and *Ureaplasma parvum* in the vagina (^*^P < 0.05) as shown in Figure 3. These results suggest that specific microbial groups may be associated with pregnancy outcomes.

### 3.4 Differences in groupings and microbiota distributions

**Figure 4:**
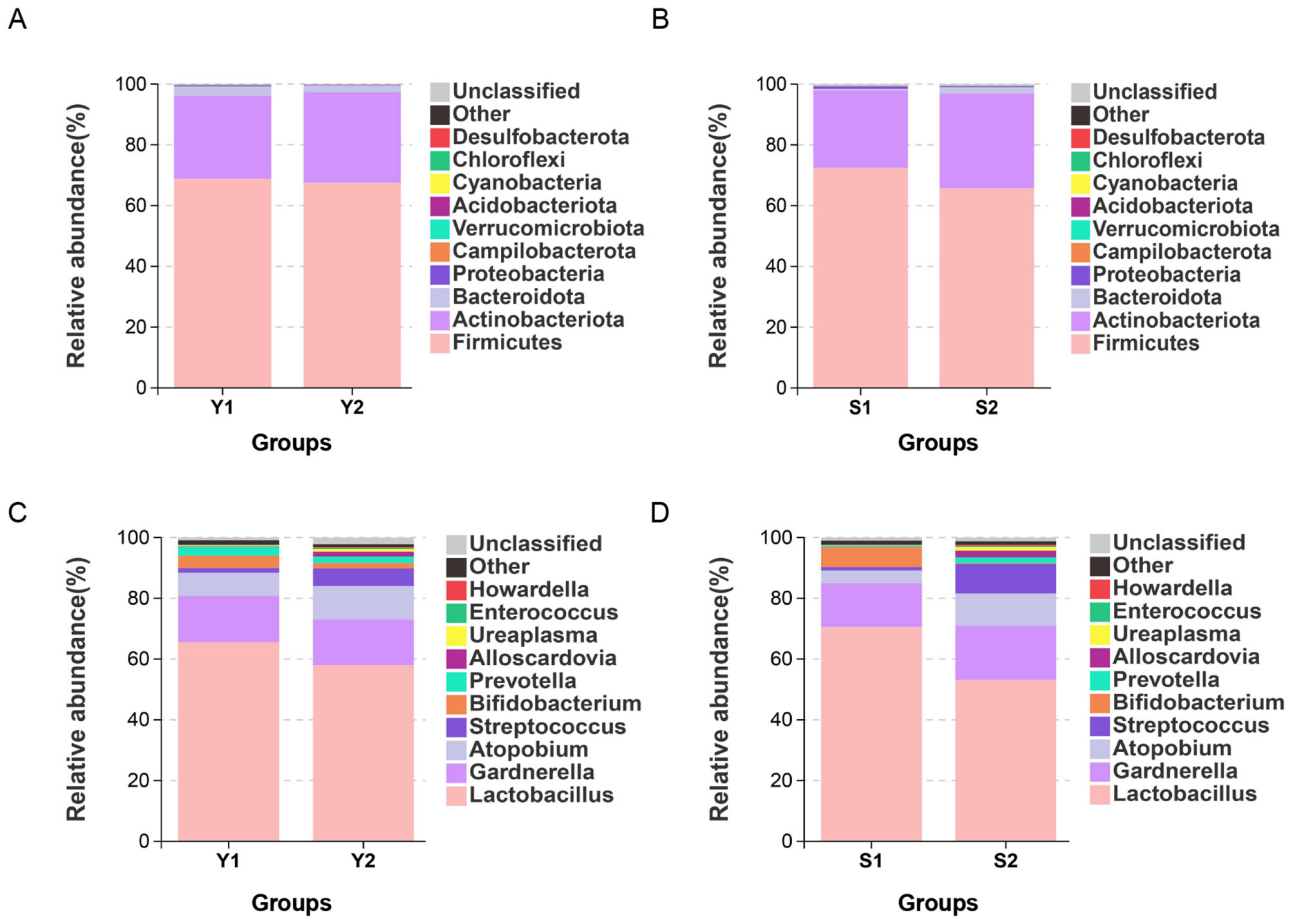
Distribution disparities among different groups and microbial communities. **(A)** Vaginal microbiota at the phylum level; **(B)** cervical microbiota at the phylum level; **(C)** vaginal microbiota at the genus level; **(D)** cervical microbiota at the genus level.

At the phylum level, *Firmicutes, Actinobacteriota, Bacteroidota* were the most common microbial microbiota in the vaginal cervix of patients with endometriosis and infertility. *Bacteroidota* and other components, see Figures 4A, B. At the genus level, the top five bacterial genera by relative abundance were: The distribution of vaginal microbiota in the successful pregnancy group was mainly *Lactobacillus, Gardnerella, Atopobium, Bifidobacterium*, and *Prevotella*. The distribution of vaginal microbiota in the transplant failure group was mainly *Lactobacillus, Gardnerella, Atopobium, Streptococcus*, and *Prevotella* (Figure 4C). The distribution of cervical microbiota in the successful pregnancy group was mainly *Lactobacillus, Gardnerella, Bifidobacterium, Atopobium* and *Streptococcus*. The distribution of cervical microbiota in the transplant failure group was mainly *Lactobacillus, Gardnerella, Atopobium, Streptococcus* and *Alloscardovia*, as shown in Figure 4D. *Lactobacillus* and *Bifidobacterium* were more abundant in vagina and cervix in the successful group than in the failed group, while*, Streptococcus, Atopobium Streptococcus* and *Prevotella* more enriched in the transplant failure group. Among them, *Gardnerella* was more abundant in the cervical group of patients with transplant failure, but more abundant in the vaginal group of patients with successful pregnancy.

### 3.5 The stability and individuality of specific taxonomic groups in the microbiomes of different body parts

**Figure 5:**
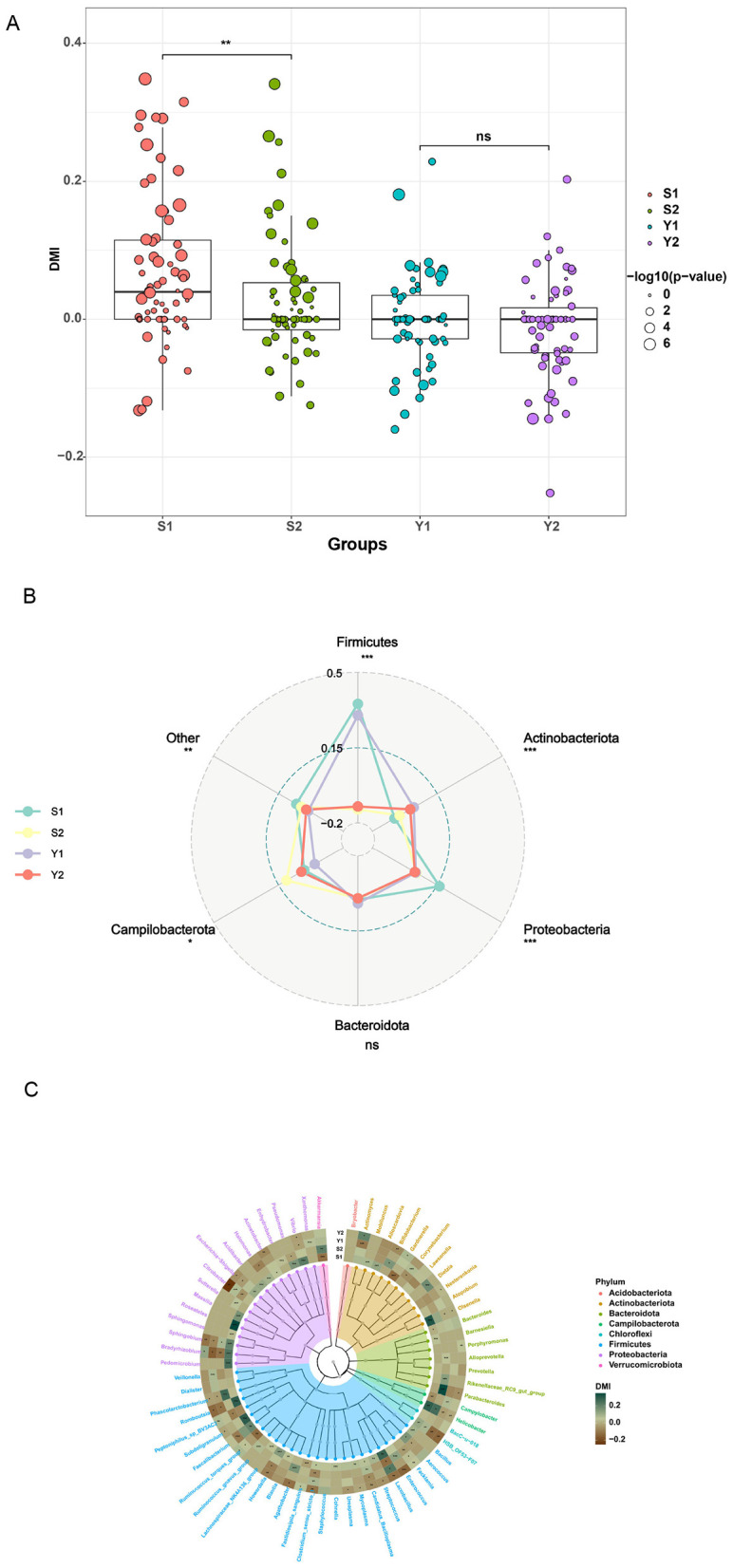
There are significant individual differences in the microbiota of different bacterial genera and the vaginal and cervical microbiota. **(A)** DMI scores of the vagina and cervix; **(B)** Radar chart of average DMI classified by site and phylum; **(C)** DMI lineage diagram of the vagina and cervix.

In the study on the stability of the microbiota at the genus level in different anatomical parts of females, we established an index named “Degree of microbial individuality (DMI)” for each microbial genus. This parameter quantitatively assesses the individual specificity of a particular microbial genus by comparing the similarity differences within individuals and among the population. The DMI value is positively correlated with the individual specificity of the microbial genus. The research data indicate that the DMI value of the cervical microbiota is significantly higher than that of the vaginal group (*P* < 0.01) and is closely associated with pregnancy outcomes. The cervical DMI value of patients with successful pregnancy is particularly prominent (Figure 5A), suggesting that the cervical microecology may play a crucial role in embryo implantation and pregnancy maintenance. In the analysis of the microbiota composition, the change in the DMI value of Firmicutes is the most significant, indicating that this microbial group may have an important regulatory effect on successful pregnancy (Figure 5B). Additionally, the DMI values of the microbiota show significant differences in different anatomical parts. For example, the DMI values of *Dietzia* in the vaginal samples and *Roseateles* in the cervical samples of patients with successful pregnancy increase significantly, suggesting that specific microbial groups may have site–dependent functional characteristics. Notably, the overall DMI level of the vagina is significantly lower than that of the cervix (*P* < 0.01), indicating that the imbalance of the cervical microbiota may have more predictive value for pregnancy outcomes than that of the vaginal microbiota (Figure 5C).

### 3.6 PICRUSt community function prediction

**Figure 6:**
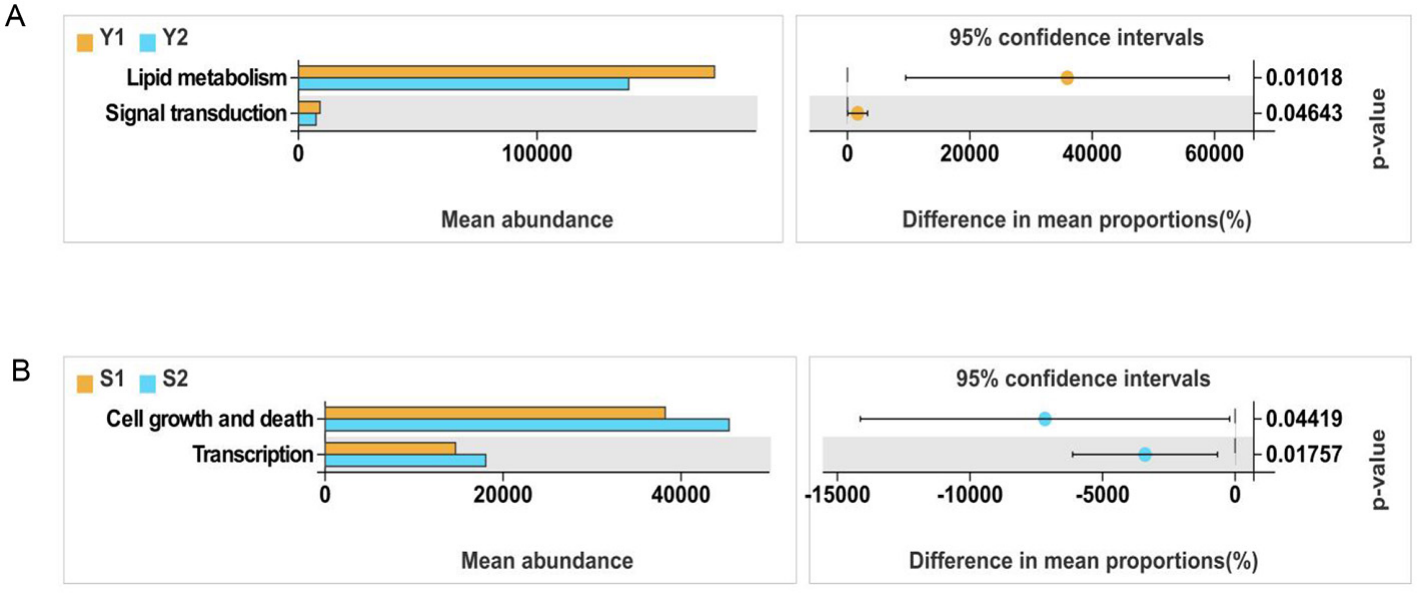
PICRUSt community function prediction. **(A)** Vaginal microbiota; **(B)** cervical microbiota.

PICRUSt functional prediction of the microbial community in all samples was predicted by PICRUSt, and KEGG metabolic pathways at three levels (levels 1–3) were obtained. The results of intra-group differential pathway *t*-test at Level 2 showed that the differential pathways in the vagina of the two groups of patients were lipid metabolism and signal transduction, and the differential pathways in the cervix included cell growth and death, and transcription, as shown in Figure 6.

### 3.7 River accumulation diagram

**Figure 7:**
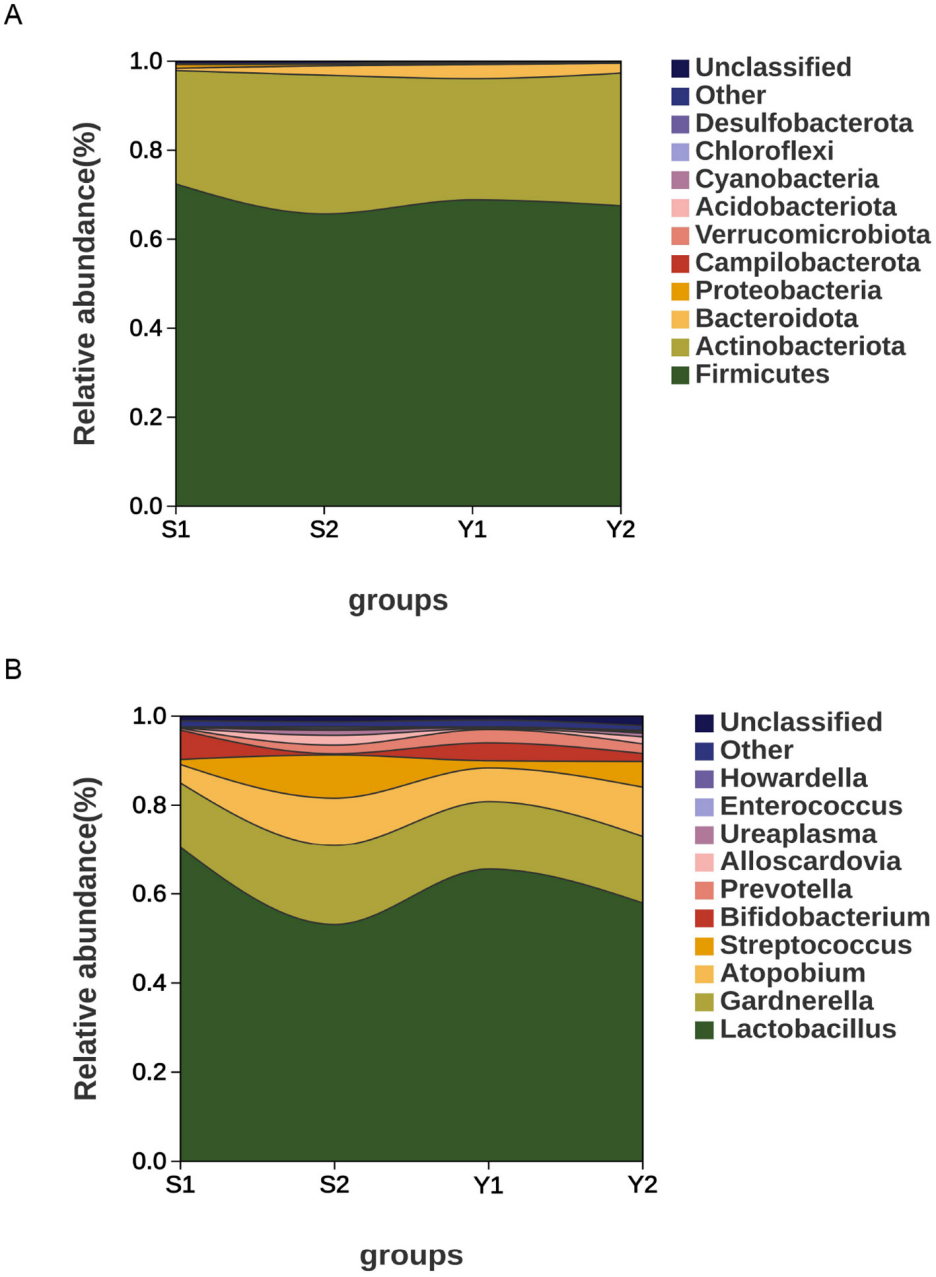
River distribution of different groups and microbiota. **(A)** Phylum level; **(B)** genus level.

*Lactobacillus* was dominant in vaginal and cervical microbiota of both successful pregnancy and transplant failure. However, *Lactobacillus* was more enriched in patients with successful pregnancies, suggesting that *Lactobacillus* may play a key role in maintaining reproductive tract health and promoting successful pregnancy. High abundance ratios of the genus lactobacillus may help inhibit the growth of harmful bacteria, thus maintain genital tract micro ecological balance. Figure 7 shows that vaginal and cervical microbiota of patients with successful pregnancy and transplant failure correspond to each other, reflecting the continuity of vaginal and cervical microbiota.

## 4 Discussion

This study provides novel insights into the cervicovaginal microbial continuum and its association with embryo implantation success in endometriosis-related infertility. Our findings highlight that cervical microbiota composition and stability, rather than vaginal diversity alone, are more strongly associated with FET outcomes. Specifically, successful pregnancies were characterized by low cervical α-diversity and dominance of *Lactobacillus* and *Bifidobacterium*, whereas failures exhibited enrichment of *Gardnerella, Atopobium*, and *Streptococcus*.

These results align with previous observations that a *Lactobacillus*-dominated, low-diversity microbiome supports mucosal homeostasis through lactic acid production, maintenance of acidic pH, and inhibition of opportunistic pathogens (13–15). Conversely, dysbiosis featuring anaerobic overgrowth may trigger chronic inflammation and impair epithelial tight-junction integrity, thereby compromising implantation (16, 17).

Our use of the degree of microbial individuality (DMI) analysis further revealed that cervical microbial stability, particularly within the phylum Firmicutes—is a signature of successful implantation. A higher DMI in cervical samples suggests that a stable, individual-specific microbial signature may facilitate immune tolerance and endometrial receptivity, consistent with prior studies demonstrating that microbial stability, not just diversity, predicts reproductive success (18).

From a mechanistic perspective, the enrichment of *Lactobacillus* and *Bifidobacterium* in successful cases may promote a favorable immune milieu via increased lactic acid and indole-3-lactic acid production, both of which modulate the Treg/Th17 balance and inhibit pro-inflammatory cytokines such as IL-6 and TNF-α. In contrast, *Gardnerella* and *Atopobium* species are known to produce sialidases and biofilms that disrupt mucosal integrity and upregulate inflammatory chemokines (e.g., IL-8), creating a cytokine-rich environment detrimental to embryo implantation.

Functionally, our PICRUSt analysis identified alterations in amino acid and carbohydrate metabolism pathways among failure-associated communities. Such metabolic perturbations may influence local nutrient availability, oxidative stress balance, and mucosal immune regulation—all critical to embryo-endometrium crosstalk. Previous metabolomic studies in ART cycles have shown that abnormal amino acid metabolism, particularly of arginine and tryptophan, correlates with implantation failure. Therefore, the present findings provide a microbiota-based metabolic rationale for these observations.

Clinically, cervical microbiota profiling could serve as a minimally invasive biomarker for endometrial receptivity assessment, complementing current morphological and molecular evaluations. Moreover, targeted modulation of cervicovaginal microbiota—through probiotics, vaginal microbiota transplantation (VMT), or prebiotic formulations—might represent a novel adjunctive strategy to enhance FET success rates in endometriosis patients.

Nevertheless, this study is limited by its small sample size and cross-sectional design, which precludes causal inference. Future longitudinal and multi-omics studies integrating metagenomics, metabolomics, and host immune profiling are warranted to confirm causality and elucidate microbial-host metabolic networks underlying implantation competence.

In conclusion, our results underscore that the cervicovaginal microbiome operates as a dynamic, continuous ecosystem, where cervical microbial stability—dominated by *Lactobacillus*—serves as a key determinant of successful embryo implantation in endometriosis-associated infertility. This integrative microbial assessment may open new avenues for individualized reproductive microbiome diagnostics and therapies.

## 5 Limitations and future directions

We acknowledge several important limitations that are inherent to this pilot investigation and that qualify the interpretation of our findings. First and foremost, the modest sample size, though justified for an initial exploratory study in a homogeneous cohort, limits the generalizability of our results and the power to detect small-effect associations. Our findings must be validated in a larger, independent cohort.

Second, the potential for contamination in low-biomass microbiome studies is a critical concern. Despite employing rigorous bioinformatic decontamination protocols (e.g., the decontam package), the lack of negative control samples (sterile swabs processed alongside patient samples) prevents definitive distinction between true biological signal and background contamination. This is particularly relevant for the low-abundance, environmentally-associated taxa reported. We strongly recommend that future work in this area prioritizes the inclusion of such controls.

Finally, 16S rDNA sequencing provides genus-level resolution; future work incorporating shotgun metagenomics and metabolomics will be essential to elucidate species-level dynamics and functionally relevant metabolites. Mechanistic studies, including *in vitro* co-culture models of endometrium and embryo with defined microbial communities, are ultimately required to dissect causal relationships.

In the next step, a prospective intervention study should be designed to examine whether correcting the microbiota based on these signatures can effectively improve pregnancy outcomes, which would be the ultimate test of its clinical relevance.

## Data Availability

The datasets presented in this study can be found in online repositories. The names of the repository/repositories and accession number(s) can be found in the article/supplementary material.

## References

[1] Chinese Chinese Society of Gynecological Surgeons Chinese Chinese Society of Obstetrics and Gynecology Endometriosis Collaboration Group. Guidelines for the diagnosis and management of endometriosis (3rd edition). Chin J Obstet Gynecol. (2021) 56:812–24. doi: 10.3760/cma.j.cn112141-20211018-00603

[2] Horne AW Zhang L. Treatment strategies of endometriosis related infertility. J Reprod Med. (2018) 27:5. doi: 10.3969/j.issn.1004-3845.2018.08.023

[3] Endometriosis combined with infertility - surgery or assisted reproduction? Int J Reprod Health/Family Plan. (2016) 35:5.

[4] Johnson NP Hummelshoj L Adamson GD Keckstein J Taylor HS Abrao MS . World endometriosis society consensus on the classification of endometriosis. Hum Reprod. (2017) 32:315–24. doi: 10.1093/humrep/dew293 27920089

[5] Yu EH Joo JK. Commentary on the new 2022 European society of human reproduction and embryology (ESHRE) endometriosis guidelines. Clin Exp Reprod Med. (2022) 49:219–24. doi: 10.5653/cerm.2022.05603 36482496 PMC9732073

[6] Babu G Singaravelu BG Srikumar R Reddy SV Kokan A. Comparative study on the vaginal flora and incidence of asymptomatic vaginosis among healthy women and in women with infertility problems of reproductive age. J Clin Diagn Res. (2017) 11:DC18–22. doi: 10.7860/JCDR/2017/28296.10417 28969122 PMC5620762

[7] Fanchin R Harmas A Benaoudia F Lundkvist U Olivennes F Frydman R. Microbial flora of the cervix assessed at the time of embryo transfer adversely affects *in vitro* fertilization outcome. Fertil Steril. (1998) 70:866–70. doi: 10.1016/S0015-0282(98)00277-5 9806568

[8] Haahr T Humaidan P Elbaek HO Alsbjerg B Laursen RJ Rygaard K . Corrigendum to: vaginal microbiota and *in vitro* fertilization outcomes: development of a simple diagnostic tool to predict patients at risk of a poor reproductive outcome. J Infect Dis. (2020) 221:1565–6. doi: 10.1093/infdis/jiz637 31968099

[9] Eslami M Naderian R Ahmadpour A Shushtari A Maleki S Mohammadian P . Microbiome structure in healthy and pregnant women and importance of vaginal dysbiosis in spontaneous abortion. Front Cell Infect Microbiol. (2025) 14:1401610. doi: 10.3389/fcimb.2024.1401610 40046910 PMC11881085

[10] Jiang I Yong PJ Allaire C Bedaiwy MA. Intricate connections between the microbiota and endometriosis. Int J Mol Sci. (2021) 22:5644. doi: 10.3390/ijms22115644 34073257 PMC8198999

[11] Harrigan JJ Abdallah HO Clarke EL Oganisian A Roy JA Lautenbach E . Respiratory microbiome disruption and risk for ventilator-associated lower respiratory tract infection. Clin Infect Dis. (2022) 74:1564–71. doi: 10.1093/cid/ciab678 34363467 PMC9630883

[12] France MT Ma B Gajer P Brown S Humphrys MS Holm JB . VALENCIA: a nearest centroid classification method for vaginal microbial communities based on composition. Microbiome. (2020) 8:166. doi: 10.1186/s40168-020-00934-6 33228810 PMC7684964

[13] Tabatabaei N Eren AM Barreiro LB Yotova V Dumaine A Allard C . Vaginal microbiome in early pregnancy and subsequent risk of spontaneous preterm birth: a case-control study. BJOG. (2019) 126:349–58. doi: 10.1111/1471-0528.15299 29791775

[14] Zhang X Zhai Q Wang J Ma X Xing B Fan H . Variation of the vaginal microbiome during and after pregnancy in Chinese women. Genomics Proteomics Bioinformatics. (2022) 20:322–33. doi: 10.1016/j.gpb.2021.08.013 35093602 PMC9684158

[15] Jonduo ME Vallely LM Wand H Sweeney EL Egli-Gany D Kaldor J . Adverse pregnancy and birth outcomes associated with *Mycoplasma hominis, Ureaplasma urealyticum* and *Ureaplasma parvum*: a systematic review and meta-analysis. BMJ Open. (2022) 12:e062990. doi: 10.1136/bmjopen-2022-062990 36028274 PMC9422885

[16] Oh KY Lee S Park J Park MH Jeong JH Yang JB . Vaginal microbiota of pregnant women with *Ureaplasma urealyticum* and *Mycoplasma hominis* infections. Front Cell Infect Microbiol. (2024) 14:1445300. doi: 10.3389/fcimb.2024.1445300 39315333 PMC11417019

[17] Freitas AC Bocking A Hill JE Money DM VOGUE Research Group. Increased richness and diversity of the vaginal microbiota and spontaneous preterm birth. Microbiome. (2018) 6:117. doi: 10.1186/s40168-018-0502-8 29954448 PMC6022438

[18] Chen C Song X Wei W Zhong H Dai J Lan Z . The microbiota continuum along the female reproductive tract and its relation to uterine-related diseases. Nat Commun. (2017) 8:875. doi: 10.1038/s41467-017-00901-0 29042534 PMC5645390

